# Lifestyle and in vitro fertilization: what do patients believe?

**DOI:** 10.1186/s40738-016-0026-5

**Published:** 2016-10-12

**Authors:** Brooke V. Rossi, Leah Hawkins Bressler, Katharine F. Correia, Shane Lipskind, Mark D. Hornstein, Stacey A. Missmer

**Affiliations:** 1 Department of Obstetrics, Gynecology and Reproductive Biology, Brigham and Women’s Hospital and Harvard Medical School, Boston, MA USA; 2 Channing Division of Network Medicine, Department of Medicine, Brigham and Women’s Hospital and Harvard Medical School, Boston, MA USA; 3 Department of Epidemiology, Harvard School of Public Health, Boston, MA USA; 4 University Hospitals Fertility Center, Kathy Risman Pavilion, Suite 310, 1000 Auburn Dr, Beachwood, OH 44122 USA

**Keywords:** Infertility, Lifestyle factors, Assisted reproductive technology (ART), In vitro fertilization (IVF)

## Abstract

**Background:**

Patients have many beliefs regarding lifestyle factors and IVF outcomes.

**Methods:**

Observational study of 208 IVF patients at an academic infertility center. Main outcome measures were perceived influence of various lifestyle factors assessed by multivariable logistic regression and *p*-value tests for linear trend (P_t_).

**Results:**

A majority of participants believed that there were many women’s lifestyle choices that were influential, compared to fewer male factors (cessation of tobacco (72 %), alcohol (69 %), caffeine (62 %), and use of vitamins (88 %)). Compared to participants with less education, participants with a higher education level were less likely to believe vitamins were helpful and some alcohol use was not harmful. As income decreased, participants were less likely to consider dietary factors contributory to IVF success, such as women (p-trend, *p* = 0.02) and men (p-trend, *p* = 0.009) consuming a full-fat dairy diet. Participants’ beliefs were most commonly influenced by physicians (84 %) and the internet (71 %).

**Conclusions:**

Patients believed many lifestyle factors are associated with IVF success. Understanding patients’ assumptions regarding the effect of lifestyle factors on IVF success may better allow physicians to counsel patients about IVF outcomes.

**Electronic supplementary material:**

The online version of this article (doi:10.1186/s40738-016-0026-5) contains supplementary material, which is available to authorized users.

## Background

Several of the most influential factors on in vitro fertilization (IVF) success, such as age, are non-modifiable. Patients and providers, however, are interested in the effects of modifiable risk factors, such as lifestyle, on the success of IVF. There is considerable evidence that modifiable factors, like smoking and weight, have a negative effect on IVF [1, 2]. Smoking negatively affects several outcomes and parameters in the IVF cycle and is associated with an increased risk of not conceiving [3–6]. In addition, body mass index (BMI) may affect a woman’s chance of successful of infertility treatment, as fecundity was found to be lower in underweight and obese women undergoing IVF compared to those with normal body weight [7]. Further, obese women were more likely to have IVF cycle cancellation, lower pregnancy, and live birth rates [8–12]. Overweight men also had a lower likelihood of pregnancy compared to men of a normal weight [13].

The effects of other factors such as psychological stress, caffeine, activity level, and environmental pollutants are less well-defined [1, 2, 14]. A prospective study of stress and IVF found that higher scores on positive affect scales were associated with a 7 % lower risk of not having a live birth and lowering stress with group intervention helped pregnancy rates [15, 16]. Conversely, general anxiety and anxiety scores were not associated with IVF outcomes, such as live birth [17]. In regards to exercise, the associations are complex. While Morris et al. showed that women who exercised more than 4 h per week had a decreased likelihood of live birth [18], a more recent study demonstrated that those with higher active living and exercise/sports indices in the past year were more likely to have a clinical pregnancy [19].

There are many studies examining IVF and different lifestyle factors, but there exists a lack of data assessing patients’ knowledge and perception of which factors are truly associated with outcomes. Previous investigations demonstrate a poor understanding of the medical issues surrounding infertility and the chance of successful IVF [20]. For example, women were unable to identify which factors have an impact on fertility [21, 22].

Finally, there are minimal data on the sources of the information and other characteristics that inform patient beliefs regarding lifestyle factors. In a study of infertility patients, participants felt that information-gathering and lifestyle change led to successful infertility treatment. Furthermore, some felt empowered by taking part in an activity they felt would impact their infertility [23]. However, after failure to conceive, others felt that their lack of lifestyle change was to blame for their infertility. To obtain information about lifestyle factors, infertility patients used the internet or books, and most spent hours on the internet. Nearly one-half of infertility patients use the internet for fertility related information [24]. Women, especially those over 35, were more likely to be influenced by on-line health information when seeking treatment.

No randomized controlled trials exist evaluating the effects of preconception advice regarding lifestyle factors on fertility outcomes in people who may have infertility [25]. One may hypothesize, however, that when patients are aware of how lifestyle factors may influence their reproductive outcome, they may be more motivated to make lifestyle changes that promote IVF success [1]. If patients are given health information, their behaviors may become healthier, as has been the case with infertile smokers [26].

In vitro fertilization is a resource-intensive treatment, often requiring a significant investment of time, money and emotional energy. Any lifestyles changes that could contribute to success, while reducing these burdens, would be significant. Our study aim was to determine which modifiable lifestyle factors patients believe to be associated with IVF success. Our primary hypothesis was that patients consider many modifiable lifestyle factors to be influential on IVF outcome. Our secondary hypotheses were that patients’ beliefs may vary based on demographics.

## Methods

We used a cross-sectional survey to assess the perceived impact of lifestyle behaviors of couples undergoing IVF. We asked subjects their opinion on several lifestyle factors (Appendix 1). Due to the large number of variables assessed, the results of the study were divided into 2 manuscripts; the current study and Hawkins et al. [27]. Couples presenting for fresh IVF cycles from 2011 through 2012 were screened for inclusion. English-speaking, heterosexual infertile couples undergoing a fresh, autologous IVF cycle with day 3 embryo transfer (cleavage transfer was standard at our institution at that time) were included. Enrolled, consenting participants were then asked questions on basic demographic information and medical history, as collected in prior studies in the IVF population [28]. We excluded donor oocyte, donor sperm, gestational carrier, and pre-implantation genetic diagnosis (PGD) cycles due to concern for subgroup heterogeneity and variability in the timing of embryo transfer. The protocol was approved by the Partners Human Research Committees. All patients were provided information on the study and completed online waivers of consent prior to beginning the survey.

Couples were screened for inclusion at the time of oocyte retrieval. They received an information packet including a one-page summary of the study and a separate document for interested participants to supply their and their partners’ email addresses. Patients who provided an email address(es) were subsequently emailed a link to the on-line survey and a login. This link directed participants to supply their login and a password to confirm eligibility.

Participants were considered enrolled after accessing the on-line study (Additional file 1). Consent was posted at the beginning of the survey. To ensure embryo transfer had occurred but serum pregnancy test had not at the time of survey completion, participants were allowed to supply responses to the survey only within a strict 11-day time window beginning three days after their egg retrieval and ending at the time of serum pregnancy test, 14 days after egg retrieval. Participants were emailed links to the survey the day of embryo transfer and before beginning the survey, participants were asked if they had undergone embryo transfer for this cycle and were denied access to the survey if they had not. To prevent responses submitted after serum pregnancy test, surveys were scheduled to expire 14 days after oocyte retrieval.

The remainder of the survey was presented with a Likert-type rating scale and included questions regarding participants’ perceived impact (very harmful, harmful, no effect, helpful, very helpful) of various lifestyle behaviors. For each of these questions, respondents were also provided the option of answering I don’t know/no opinion. The surveys were designed specifically for this study. The list of behaviors was created by considering: 1), the current evidence and literature on lifestyle factors and IVF and 2) feedback from IVF providers who reported their patients’ modification or questions about lifestyle factors and IVF. After answering these questions with regard to the perceived impact of their behavior, participants were then asked to answer these questions with regard to the perceived impact of their partner’s behavior. Lastly, participants were asked if a number of sources contributed to their beliefs about lifestyle (physician, nurse, book, internet, friends, family, and partner).

**Fig. 1:**
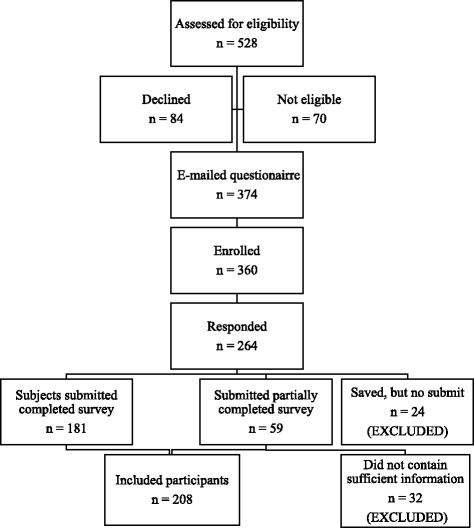
Subject recruitment and outcome

Participant responses were restricted by IP address to ensure participants did not complete the survey multiple times. Couples were not excluded if only one partner provided their email address. To comply with Institutional Review Board regulations regarding de-identification of participants completing online surveys through a third party, partners’ survey responses were not linked by couple. Responses were considered for inclusion in analysis from all submitted questionnaires in which a participant answered all demographic questions and at least part of the questions on lifestyle behaviors. Respondents who saved but did not submit responses, even in cases where participants completed the entire survey, were excluded from analysis due to concern that a participant’s non-submission represented indecision or discomfort with sharing responses. Responses from a total of 208 participants (45 % of invited, eligible participants) were included in analysis. Subject recruitment and outcomes are in Fig. 1.

SAS 9.2 (SAS Institute Inc., Cary, NC) was used for all data analysis. Dichotomous outcomes were consolidated for analysis; helpful and very helpful versus harmful and very harmful. We excluded neutral responses, missing responses, and “no response/I don’t know” replies. All variables in Table 1 were considered and gender, age, and education level were found to be confounders. Multivariable logistic regression analyses adjusting for gender, age, and education were performed to estimate the effects of explanatory variables such as gender, infertility diagnosis, and duration of infertility on perceptions about lifestyle behaviors. Results are presented as adjusted odds ratios (OR) and 95 % confidence intervals (CI). Wald p-value tests for linear trend (for which statistical significance was assumed for *p* < 0.05).

**Table 1:** Demographics of 208 IVF patients

Characteristic	Women	Men
*N* (%)	138 (66.3 %)	70 (33.7 %)
Age		
< = 34	48 (34.8 %)	20 (28.6 %)
35–37	37 (26.8 %)	14 (20.0 %)
38–40	31 (22.5 %)	16 (22.9 %)
41–42	16 (11.6 %)	7 (10.0 %)
43+	6 (4.3 %)	13 (18.6 %)
Education		
High school or 2-year college	17 (22.3 %)	9 (12.8 %)
4-year college	40 (29.0 %)	21 (30.0 %)
Master’s degree	44 (31.9 %)	26 (37.1 %)
MD/PhD/JD	37 (26.8 %)	14 (20.0 %)
Annual household income		
< $100,000	25 (18.3 %)	17 (24.3 %)
$100,001–$150,000	43 (31.4 %)	20 (28.6 %)
150,001–$200,000	27 (19.7 %)	13 (18.6 %)
> $200,001	42 (30.6 %)	20 (28.5 %)
Race/Ethnicity (all that apply)		
Asian, Pacific Islander or other Asian	15 (11.1 %)	9 (12.8 %)
Caucasian	107 (78.7 %)	48 (68.6 %)
Other	13 (12.5 %)	13 (18.6 %)
Number of months trying to get pregnant		
< 6 months	2 (1.5 %)	8 (11.4 %)
6–12	20 (14.6 %)	9 (12.9 %)
13–24	46 (33.6 %)	28 (40.0 %)
> 24 months	69 (50.4 %)	25 (35.7 %)
Primary infertility diagnosis (all that apply)		
Ovulation	14 (10.1 %)	6 (8.6 %)
Blocked tubes	23 (16.7 %)	4 (5.7 %)
Uterine factor	2 (1.4 %)	0 (0 %)
Endometriosis	10 (7.2 %)	5 (7.1 %)
Male factor	29 (21.0 %)	13 (18.6 %)
DOR	35 (25.4 %)	15 (21.4 %)
Unexplained	51 (37.0 %)	24 (34.3 %)
Don’t know	4 (2.9 %)	5 (7.1 %)
Other Etiology	10 (7.2 %)	10 (14.3 %)

## Results

There were 208 participants, consisting of 138 (66.3 %) women and 70 (33.7 %) men, mostly age 35 or older and educated, with a variety of infertility diagnoses (Table 1). Due to the large amount of data collected and manuscript length limitations, we will only present data for the factors most respondents found important.

In regards to women, greater than 71.7 % of participants felt that women not smoking was helpful to the success of the IVF cycle, and 86.1 % believed daily second-hand smoke exposure was harmful. A majority of participants felt that avoiding alcohol (69.2 %) and caffeine (62.1 %) were helpful. Overall, vitamins were considered beneficial, including prenatal vitamins (88.3 %), multivitamins (81.6 %), vitamin C (66.1 %), vitamin E (65.2 %), and vitamin D (68.1 %). Most participants felt that an increase in fruits and vegetables (83.7 %) and an organic diet (57.6 %) was helpful; otherwise there were no consistent beliefs concerning diet or artificial sweeteners. Finally, many respondents felt that ibuprofen (74.7 %) and cold/allergy medicine (70.9 %) were harmful, and that Tylenol (79.0 %) use had no effect on IVF cycle success.

Considering men, 68.1 % felt not smoking was helpful and 76.0 % felt that daily second-hand smoke exposure was harmful. Most (65.4 %) considered no alcohol use helpful. However, when asked specifically about 1–4 drinks of beer, wine, or liquor per week, a majority believed this amount of alcohol to have no effect. Conversely, a majority felt >4 drinks of beer, wine, or liquor per week were harmful to IVF cycle success. Sixty-five percent felt that male use of multivitamins was helpful, but no other vitamin use was thought to be influential on IVF cycle outcome. Participants did not believe diet, caffeine, artificial sweeteners, or over-the-counter medicines were either helpful or harmful.

**Fig. 2:**
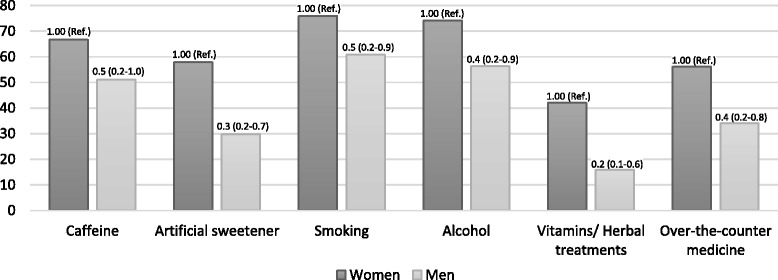
Percent of women and men participants who believed that women avoiding these factors were helpful to IVF cycle success. Odd ratio and 95 % CI reported. Multivariate model includes gender, age (ordinal), and education (ordinal) in addition to the primary predictor

**Fig. 3:**
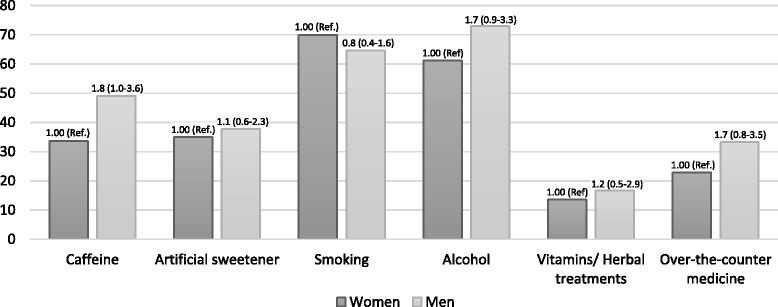
Percent of women and men participants who believed that men avoiding these factors were helpful to IVF cycle success. Odd ratio and 95 % CI reported. Multivariate model includes gender, age (ordinal), and education (ordinal) in addition to the primary predictor

In general, participants felt that their own lifestyle choices were more influential than their partners believed them to be (Figs. 2 and 3). For example, compared to women, men were less likely to think that it was helpful for women to avoid artificial sweetener (OR 0.3; CI 0.2–0.7), smoking (OR 0.5; CI 0.2–0.9), alcohol (OR 0.4; CI 0.2–0.9), vitamins/herbal treatments (OR 0.2; 0.1–0.6), or over-the-counter medications (OR 0.4; CI 0.2–0.8). Similarly, compared to women, men were more likely to believe that it was helpful for a man to avoid caffeine (OR 1.8; CI 1.0–3.6), alcohol (OR 1.7; CI 0.9–3.3), or over-the-counter medications (OR 1.7; CI 0.8–3.5) (all not statistically significant).

Associations among the lifestyle factors and demographics were assessed. Education level and income were significantly associated with beliefs. Both men and women with doctorate degrees had approximately 30 % lower odds of believing vitamins were helpful or alcohol and caffeine were harmful. Trends were also seen among income, as men and women with a decreased income were less likely to feel that low carbohydrate, high protein, full-fat dairy diets were helpful to IVF success (all *p*_*t*_ <0.05). Finally, when asked which sources led to their beliefs, participants felt physicians (84 %) and nurses (83 %) were influential or very influential [28]. Participants also considered the internet (71 %), books (65 %), and family (53 %) influential or very influential. In general, women considered more sources influential than men.

## Discussion

The present work characterizes patients’ beliefs surrounding lifestyle factors and IVF. A majority of patients did accurately recognize the harm of personal cigarette use and second-hand smoke exposure [3, 6, 29, 30]. However, in general, patient beliefs were inconsistent with the current evidence surrounding lifestyle factors and IVF outcomes. There was concern for some amount of alcohol and caffeine, but they did not recognize that a minimal amount of either of these may be harmful [31–33]. Also, many patients considered certain lifestyle factors influential even though existing evidence is not strongly supportive of an association (vitamins, organic diet) [34]. While this over-cautiousness may not be harmful from a medical standpoint, we do need to consider the stress and financial impact of changing behaviors or lifestyles without scientific support. These overall beliefs measure physician success at patient education and highlight existing knowledge deficits.

One of the unique characteristics of this study is the assessment of the each member’s own lifestyle factors, as well as their beliefs about their partner’s lifestyle factors and IVF outcomes. In general, we observed that both men and women believed that their own gender’s lifestyle factors and behaviors were more influential than the opposite-sex’s behaviors. Patients may truly believe, based on what they have heard or learned, that their gender’s lifestyle factors are more important than their partners. It is also possible, however, that this finding suggests that patients are practicing self-blame. Self-blame is one of the coping mechanisms exhibited by infertile men and women, and this coping mechanism may be further increase their distress [35]. However, men and women manage the stress of infertility in different ways [36]. Women may be more likely to self-blame as a coping strategy [37]. This may explain that we had more significant variables for women’s’ beliefs of themselves than men’s. Better education of patients may help them understand which lifestyle factors may or may not have an impact and which are under their control. This may reduce self-blame and therefore reduce distress.

We noted several patient characteristics which correlated with beliefs, namely education and income level. The differences in education among patients using IVF are evident in many facets of infertility treatment, from attainment of care to beliefs. A higher education level (post-secondary degree) has been associated with a greater likelihood of having an infertility evaluation and treatment [38]. A survey of national infertility treatment use demonstrated that while 21–23 % of women aged 25–44 have used infertility treatment, only 10 % of women with a high-school diploma had treatment [39]. Our study demonstrated that participants with higher education believed vitamins were more influential, but did not feel that other habits, such as alcohol or caffeine were associated with IVF success. The potential effect of education level on IVF has not been extensively studied, however, one study did not demonstrate an association between education level and live birth rate [40]. Thus, the differences in lifestyle beliefs may lead patients to practice different behaviors or use different therapies (i.e. vitamins), but these changes may not influence IVF outcomes.

Similar trends were seen with income. As with education level, Chandra et al. also determined that infertility treatment was more prevalent for those with a household income above the poverty level (21 %) compared to below the poverty level (13 %) [39]. Even in a state with mandated IVF and infertility coverage, over 60 % had a household income of > $100,000 [41]. There are no studies evaluating the association between income level and IVF success. We found that those at a lower income level were more likely to incorrectly believe that certain dietary factors (alcohol, fat or carbohydrate composition) influenced IVF success, when they were not strongly supported in the literature.

Many patients seek information regarding lifestyle and infertility. Although a majority of our participants felt that care providers were influential, some patients felt that the information they received from their clinician was not sufficient and they sought other information [23]. The participants also noted that many of them had already made lifestyle changes that the clinicians discussed and they were seeking additional information, including that regarding complementary and alternative medicine treatments. Finally, participants reported that information gathering was empowering and provided emotional support though connections with other patients. Our study and others show that the internet is a popular way for patient to learn about infertility and IVF. As long ago as 2003, Haagen et al. demonstrated that 66 % of infertility participants surveyed used the internet for fertility-related problems [42]. In accord with our findings, more women than men used the internet, and education and household income was associated with amount of use.

Weaknesses of this study include issues related to the diversity of the study sample and that our survey was done in a single practice. Our practice is located in an academic medical center in a large Northeast city. Of course, any study done at one center may not be representative of the general population. For example, 88 % of our participants had a college degree or higher and only 24 % had an income of < $100,000. This is significantly different than the general US population in which 28 % have a college degree or higher and the median income is approximately $53,000, consistent with a large academic northeastern city [43]. The racial make-up of our study, however, was similar to the general population and our practice is in a state with mandated insurance coverage, which may allow for more patients to have access to IVF coverage compared to other states. Only 13 % of respondents were not of Caucasian or Asian ethnicity, and 88 % had at least a 4-year college education, whereas in the United States in general, only 31 % have a 4-year college degree. While this educational level, may be not in-line with the general population, it may be more typical of the general US IVF population, which tends to have a higher education level. Another weakness includes the inability to assess if one partner’s response was correlated to the other partner’s response. Each subject was independent and not linked to their partner. Future analyses could include tracking couple’s responses together to assess for correlations. Finally, our survey response rate was 45 %, which may limit generalizability to the overall IVF population.

We were also concerned that home pregnancy testing may influence results and took steps to minimize this potential source of bias. We advised patients to wait for their scheduled serum pregnancy test and only allowed access to the survey until the date of the test to decrease the risk that premature knowledge of their cycle outcome would influence their responses.

## Conclusions

Our results demonstrate that IVF patients are aware of the established associations between lifestyle factors and cycle success. However, there is also a tendency for patients to ascribe importance to many factors that are not supported as influential by medical literature. Findings of our study may assist care providers with patient education and further help patients target their efforts towards meaningful lifestyle change. We advocate for better education about the impact of lifestyle on IVF for all, acknowledging the impact of socioeconomic status. Moreover, results highlight the need to tailor the education to appropriate sources, such as the internet, and recognize the importance of the care providers’ guidance and recommendations. Assessment of belief modification after an educational intervention about lifestyle factors and IVF outcomes is merited.

## Appendix

**Table 2:** Lifestyle factors assessed in the survey

Caffeine	
Alcohol	
Beer	
White wine	
Red wine	
Liquor	
Smoking	
Vitamins	
Prenatal vitamin	
Multivitamin	
Zinc	
Vitamin E	
Vitamin C	
Vitamin B6	
Vitamin D	
Chinese herbal medicine	
Dehydroepiandrosterone	
Over-the-counter medications	
Baby aspirin	
Ibuprofen	
Acetaminophen	
Cold/allergy medicine	
Food/Diet	
Regular diet	
Low-fat diet	
Low-carbohydrate diet	
Organic diet	
Vegetarian diet	
High protein diet	
Increase in fruits and vegetables	
Full-fat milk or dairy products	
Artificial Sweeteners	
Stress^a^	
Attitude^a^	
Negative	
Positive	
Body weight^a^	
Normal	
Underweight	
Overweight	
Obese	
Exercise^a^	
Yoga^a^	
Acupuncture^a^	
Rest^a^	
Prayer^a^	
Cell phone use^a^	
Environment^a^	
Exposure to plastics	

^a^Indicates that results are presented in Hawkins et al. [28].

## Additional file

Additional file 1: Lifestyle factors and in vitro fertilization questionnaire. (PDF 113 kb)

## Abbreviations

ART: Assisted reproductive technologies
IVF: In vitro fertilization
BMI: Body mass index
PGD: Preimplantation genetic diagnosis
OR: Odds ratio
CI: Confidence interval
