# Supplementary material for: Lifestyle and in vitro fertilization: what do patients believe?
Source: Fertil Res Pract. 2016 Oct 12;2:11. doi: 10.1186/s40738-016-0026-5 (PMC5424337; doi:10.1186/s40738-016-0026-5)
Supplement: Additional file 1: — Lifestyle factors and in vitro fertilization questionnaire. (PDF 113 kb) [file 40738_2016_26_MOESM1_ESM.pdf]

Lifestyle Factors and In Vitro Fertilization

1. Welcome and consent

You have been invited to participate in a research study called “Lifestyle Factors and the Success of In Vitro Fertilization (IVF)," led by Drs. Brooke Rossi and Stacey Missmer.

The purpose of this study is to find out which lifestyle factors or activities you think will affect the success of IVF treatment. Knowing these factors will help physicians and scientists to focus our efforts to better inform all patients about which factors are beneficial and which are not.

About 150 people will participate.

It will take about 10 minutes to complete the survey.

Your answers will be anonymous; we will not link your name or any other information about you to your answers. Also, we will not be able to trace your answers back to your computer.

Taking part is voluntary and you may stop at any time. If you decide not to participate, it won't affect your medical care you receive at Partners now or in the future, or any benefits they receive now or have a right to receive.

If you have any questions about this project, you can contact Dr. Brooke Rossi at 617-732-4841 or Dr. Stacey Missmer at 617-525-2747.

If you’d like to speak to someone not involved in this research about your rights as a research subject, or any concerns or complaints you may have about the research, contact the Partners Human Research Committee at 617-424-4100.

1. Please indicate the following:

- ◯ I AGREE TO PARTICIPATE IN THIS RESEARCH
- ◯ I DO NOT WANT TO TAKE PART IN THIS RESEARCH

2. Declined Survey- Reasons

1. Please describe your reason for not taking part in the study.

- ☐ Not interested in research
- ☐ Survey is too long
- ☐ Not interested in this topic
- ☐ Adds stress

3. Declined Survey- Age and gender

1. What is your age (in years)?

- ◯ 34 or younger
- ◯ 35-37
- ◯ 38-40
- ◯ 41-42
- ◯ 43 or older

2. Please indicate gender:

- ◯ Woman
- ◯ Man

4. Declined Survey- Demographics

1. Which best describes your ethic background?

- ◯ African American
- ◯ American Indian/ Native Alaskan
- ◯ Asian
- ◯ Caucasian
- ◯ Hispanic
- ◯ Latino
- ◯ Hispanic/ Latino
- ◯ Other Asian American/ Pacific Islander
- ◯ Other

2. What is your religious preference?

- ◯ Catholic
- ◯ Protestant
- ◯ Islam
- ◯ Judaism
- ◯ Secular/Agnosticism/Non-religious
- ◯ Buddhism
- ◯ Hinduism
- ◯ Chinese traditional
- ◯ Other

3. What is the highest education level you have completed?

- ◯ Less than high school
- ◯ High school
- ◯ 2-year college
- ◯ 4-year college
- ◯ Master’s degree
- ◯ Medical degree/ Ph.D./ Law degree

4. What is your yearly household income (before taxes)?

- ◯ Less than $50,000
- ◯ $50,001 - $100,000
- ◯ $100,001 - $150,000
- ◯ $150,001 - $200,000
- ◯ $200,001 - $250,000
- ◯ More than $250,000

5. Men- Prior pregnancies

1. How long has it been since you got your current partner pregnant?

- Less than 1 year
- 1-2 years
- 3-4 years
- 5 or more years

6. Age and gender

1. What is your age (in years)?

- 34 or younger
- 35-37
- 38-40
- 41-42
- 43 or older

2. Please indicate gender:

- Woman
- Man

7. Men's history

1. How many times have you gotten someone pregnant in the past (including through intercourse and/or infertility treatments)?

- 0
- 1
- 2
- 3
- 4
- 5 or more

8. Women's history

1. How many times have you been pregnant?

- ◯ 0
- ◯ 1
- ◯ 2
- ◯ 3
- ◯ 4
- ◯ 5 or more times

2. How many live births have you had?

- ◯ 0
- ◯ 1
- ◯ 2
- ◯ 3
- ◯ 4 or more

3. How long has it been since your last pregnancy?

- ◯ I have never been pregnant
- ◯ Less than 1 year
- ◯ 1-2 years
- ◯ 3-4 years
- ◯ 5 or more years

9. Infertility history

1. How many months have you been trying to get pregnant?

- ◯ Less than 6 months
- ◯ 6-12
- ◯ 13-24
- ◯ More than 24 months

2. What has your doctor think is the primary cause of your difficulty getting pregnant?

- ◯ Ovulation problem
- ◯ Blocked tubes
- ◯ Uterine factor
- ◯ Endometriosis
- ◯ Male factor
- ◯ Decreased ovarian function
- ◯ Unexplained
- ◯ Cannot remember or do not know the cause
- ◯ Other

3. How many IVF cycles have you done in the past (1 cycle= egg retrieval and embryo transfer)?

- ◯ 0
- ◯ 1
- ◯ 2
- ◯ 3
- ◯ 4 or more

10. IVF History

1. How many IVF cycles have you done that have resulted in the delivery of a live baby?

- ◯ 0
- ◯ 1
- ◯ 2
- ◯ 3
- ◯ 4 or more

11. Demographics

1. Which best describes your ethic background?

- ◯ African American
- ◯ American Indian/ Native Alaskan
- ◯ Asian
- ◯ Caucasian
- ◯ Hispanic
- ◯ Latino
- ◯ Other Asian American/ Pacific Islander
- ◯ Other

2. What is your religious preference?

- ◯ Catholic
- ◯ Protestant
- ◯ Islam
- ◯ Judaism
- ◯ Secular/Agnosticism/Non-religious
- ◯ Buddhism
- ◯ Hinduism
- ◯ Chinese traditional
- ◯ Other

3. What is the highest education level you have completed?

- ◯ Less than high school
- ◯ High school
- ◯ 2-year college
- ◯ 4-year college
- ◯ Master’s degree
- ◯ Medical degree/ Ph.D./ Law degree

4. What is your yearly household income (before taxes)?

- ◯ Less than $50,000
- ◯ $50,001 - $100,000
- ◯ $100,001 - $150,000
- ◯ $150,001 - $200,000
- ◯ $200,001 - $250,000
- ◯ More than $250,000

12.

The next set of questions will ask you how important you believe different lifestyle factors are to the success of your IVF cycle.

For each question that follows, please consider how important you think the use of the item or activity is to the success (getting pregnant and having a baby) of your IVF cycle.

If you do not use an item or do an activity, base your answer on how you think the item or activity might affect the IVF cycle of someone who does.

13. Caffeine

1. How do you think your use of caffeinated drinks is to the success (getting pregnant and having a baby) of your IVF cycle?

	Very harmful	Harmful	No effect	Helpful	Very helpful	I don't know/ No opinion
No drinks per week	◯	◯	◯	◯	◯	◯
1-7 drinks per week	◯	◯	◯	◯	◯	◯
More than 7 drinks per week	◯	◯	◯	◯	◯	◯

14. Artificial sweetners

1. How do you think your use artificial sweeteners affect the success (getting pregnant and having a baby) of your IVF cycle?

	Very harmful	Harmful	No effect	Helpful	Very helpful	I don't know/ No opinion
No use	◯	◯	◯	◯	◯	◯
Less than daily use	◯	◯	◯	◯	◯	◯
At least once per day	◯	◯	◯	◯	◯	◯
More than once per day	◯	◯	◯	◯	◯	◯

15. Smoking

Lifestyle Factors and In Vitro Fertilization

1. How do you think smoking the following number cigarettes affect the success (getting pregnant and having a baby) of your IVF cycle?

	Very harmful	Harmful	No effect	Helpful	Very helpful	I don't know/ No opinion
No use	◯	◯	◯	◯	◯	◯
Daily second-hand smoke	◯	◯	◯	◯	◯	◯
Less than 5 cigarettes per day	◯	◯	◯	◯	◯	◯
5-10 cigarettes per day	◯	◯	◯	◯	◯	◯
11-20 cigarettes per day	◯	◯	◯	◯	◯	◯
More than 20 cigarettes per day	◯	◯	◯	◯	◯	◯

16. Alcohol

1. How do you think the following types and amount of alcohol affects the success (getting pregnant and having a baby) of your IVF cycle?

	Very harmful	Harmful	No effect	Helpful	Very helpful	I don't know/ No opinion
No alcohol	◯	◯	◯	◯	◯	◯
1-4 drinks of beer per week	◯	◯	◯	◯	◯	◯
More than 4 drinks of beer per week	◯	◯	◯	◯	◯	◯
1-4 drinks of white wine per week	◯	◯	◯	◯	◯	◯
More than 4 drinks of white wine per week	◯	◯	◯	◯	◯	◯
1-4 drinks of red wine per week	◯	◯	◯	◯	◯	◯
More than 4 drinks of red wine per week	◯	◯	◯	◯	◯	◯
1-4 drinks of liquor per week	◯	◯	◯	◯	◯	◯
More than 4 drinks of liquor per week	◯	◯	◯	◯	◯	◯

17. Foods

Lifestyle Factors and In Vitro Fertilization

1. How do you think the following types of food affect the success (getting pregnant and having a baby) of your IVF cycle?

	Very harmful	Harmful	No effect	Helpful	Very helpful	I don't know/ No opinion
No specific (regular) diet	◯	◯	◯	◯	◯	◯
Low fat diet	◯	◯	◯	◯	◯	◯
Low carbohydrate diet	◯	◯	◯	◯	◯	◯
Vegetarian diet	◯	◯	◯	◯	◯	◯
Organic food	◯	◯	◯	◯	◯	◯
Increase in fruit and vegetables	◯	◯	◯	◯	◯	◯
High protein diet	◯	◯	◯	◯	◯	◯
Full-fat milk or dairy products	◯	◯	◯	◯	◯	◯

18. Vitamins and herbal medicine

1. How do you think that taking the following vitamins or herbs affect the success (getting pregnant and having a baby) of your IVF cycle?

	Very harmful	Harmful	No effect	Helpful	Very helpful	I don't know/ No opinion
No vitamins or herbs	◯	◯	◯	◯	◯	◯
Prenatal vitamin	◯	◯	◯	◯	◯	◯
Mulitvitamin	◯	◯	◯	◯	◯	◯
Zinc	◯	◯	◯	◯	◯	◯
Dehydroepiandrosterone (DHEA)	◯	◯	◯	◯	◯	◯
Vitamin B6	◯	◯	◯	◯	◯	◯
Vitamin C	◯	◯	◯	◯	◯	◯
Vitamin D	◯	◯	◯	◯	◯	◯
Vitamin E	◯	◯	◯	◯	◯	◯
Chinese herbal medicine	◯	◯	◯	◯	◯	◯

19. Over the counter medications

1. How do you think that taking the following over the counter medications affect the success (getting pregnant and having a baby) of your IVF cycle?

	Very harmful	Harmful	No effect	Helpful	Very helpful	I don't know/ No opinion
No over the counter medications	◯	◯	◯	◯	◯	◯
Baby aspirin	◯	◯	◯	◯	◯	◯
Ibuprofen	◯	◯	◯	◯	◯	◯
Tylenol	◯	◯	◯	◯	◯	◯
Cold/allergy medication	◯	◯	◯	◯	◯	◯

20. Stress

1. How do you think that stress affects the success (getting pregnant and having a baby) of your IVF cycle?

	Very harmful	Harmful	No effect	Helpful	Very helpful	I don't know/ No opinion
No stess	◯	◯	◯	◯	◯	◯
Stress	◯	◯	◯	◯	◯	◯

21. Attitude

1. How do you think that your attitude affects the success (getting pregnant and having a baby) of your IVF cycle?

	Very harmful	Harmful	No effect	Helpful	Very helpful	I don't know/ No opinion
Neither positive nor negative attitude	◯	◯	◯	◯	◯	◯
Positive attitude	◯	◯	◯	◯	◯	◯
Negative attitude	◯	◯	◯	◯	◯	◯

22. Body weight

1. How do you think the following different body weights affect the success (getting pregnant and having a baby) of an IVF cycle?

	Very harmful	Harmful	No effect	Helpful	Very helpful	I don't know/ No opinion
Underweight	◯	◯	◯	◯	◯	◯
Normal weight	◯	◯	◯	◯	◯	◯
Overweight	◯	◯	◯	◯	◯	◯
Obese	◯	◯	◯	◯	◯	◯

23. Yoga

1. How do you think different amounts of yoga affects the success (getting pregnant and having a baby) of your IVF cycle?

	Very harmful	Harmful	No effect	Helpful	Very helpful	I don't know/ No opinion
No yoga	◯	◯	◯	◯	◯	◯
Once per week	◯	◯	◯	◯	◯	◯
2-6 sessions per week	◯	◯	◯	◯	◯	◯
7 or more sessions per week	◯	◯	◯	◯	◯	◯

24. Accupuncture

1. How do you think accupuncture around the time of embryo transfer affects the success (getting pregnant and having a baby) of your IVF cycle?

	Very harmful	Harmful	No effect	Helpful	Very helpful	I don't know/ No opinion
No accupuncture	◯	◯	◯	◯	◯	◯
Accupuncture	◯	◯	◯	◯	◯	◯

25. Exercise

1. How do you think exercise affects the success (getting pregnant and having a baby) of your IVF cycle?

	Very harmful	Harmful	No effect	Helpful	Very helpful	I don't know/ No opinion
No exercise	◯	◯	◯	◯	◯	◯
Less than 1 hour per week	◯	◯	◯	◯	◯	◯
1-3 hours per week	◯	◯	◯	◯	◯	◯
4 or more hours per week	◯	◯	◯	◯	◯	◯

26. Rest

1. How do you think that resting (avoiding exercise or strenuous activity) the week after the embryo transfer affects the success (getting pregnant and having a baby) of your IVF cycle?

	Very harmful	Harmful	No effect	Helpful	Very helpful	I don't know/ No opinion
Normal activities/ no rest	◯	◯	◯	◯	◯	◯
Limits on strenuous activities and exercise	◯	◯	◯	◯	◯	◯
Bed rest	◯	◯	◯	◯	◯	◯

27. Prayer

1. How do you think praying for the success of your IVF cycle affects the success (getting pregnant and having a baby) of your IVF cycle?

	Very harmful	Harmful	No effect	Helpful	Very helpful	I don't know/ No opinion
No prayer	◯	◯	◯	◯	◯	◯
Prayer	◯	◯	◯	◯	◯	◯

28. Cell phones

1. How do you think that typical cell phone usage affects the success (getting pregnant and having a baby) of your IVF cycle?

	Very harmful	Harmful	No effect	Helpful	Very helpful	I don't know/ No opinion
Limiting cell phone usage	◯	◯	◯	◯	◯	◯
Typical cell phone usage	◯	◯	◯	◯	◯	◯

29. Environment/ plastics

1. How do you think typical exposure to plastics (water bottles, food containers) affects the success of your IVF cycle (getting pregnant and having a baby) of your IVF cycle?

	Very harmful	Harmful	No effect	Helpful	Very helpful	I don't know/ No opinion
Limiting exposure to plastics	◯	◯	◯	◯	◯	◯
Typical exposure to plastics	◯	◯	◯	◯	◯	◯

30. Other factors

1. If there any other lifestyle factors (items or activities) that you think affect the success of your IVF cycle that have not been mentioned in this survey, please write them here.

31. Partner lifestyle factors

The next set of questions will ask you how important you believe YOUR PARTNER'S USE of different lifestyle factors are to the success of your IVF cycle.

For each question that follows, please consider how important you think YOUR PARTNER'S use of the item or activity is to the success (getting pregnant and having a baby) of your IVF cycle.

If YOUR PARTNER does not use an item, base your answer on how you think the item might affect the IVF cycle of someone who does.

32. Caffeine- Partner use

1. How do you think your partner's use of caffeinated drinks is to the success (getting pregnant and having a baby) of your IVF cycle?

	Very harmful	Harmful	No effect	Helpful	Very helpful	I don't know/ No opinion
No drinks per week	◯	◯	◯	◯	◯	◯
1-7 drinks per week	◯	◯	◯	◯	◯	◯
More than 7 drinks per week	◯	◯	◯	◯	◯	◯

33. Artificial sweetners- Partner use

1. How do you think your partner's use artificial sweeteners affect the success (getting pregnant and having a baby) of your IVF cycle?

	Very harmful	Harmful	No effect	Helpful	Very helpful	I don't know/ No opinion
No use	◯	◯	◯	◯	◯	◯
Less than daily use	◯	◯	◯	◯	◯	◯
At least once per day	◯	◯	◯	◯	◯	◯
More than once per day	◯	◯	◯	◯	◯	◯

34. Smoking- Partner use

1. How do you think your partner's smoking the following number cigarettes affect the success (getting pregnant and having a baby) of your IVF cycle?

	Very harmful	Harmful	No effect	Helpful	Very helpful	I don't know/ No opinion
No use	◯	◯	◯	◯	◯	◯
Daily second-hand smoke	◯	◯	◯	◯	◯	◯
Less than 5 cigarettes per day	◯	◯	◯	◯	◯	◯
5-10 cigarettes per day	◯	◯	◯	◯	◯	◯
11-20 cigarettes per day	◯	◯	◯	◯	◯	◯
More than 20 cigarettes per day	◯	◯	◯	◯	◯	◯

35. Alcohol- Partner use

1. How do you think your partner drinking the following types and amount of alcohol affects the success (getting pregnant and having a baby) of your IVF cycle?

	Very harmful	Harmful	No effect	Helpful	Very helpful	I don't know/ No opinion
No alcohol	◯	◯	◯	◯	◯	◯
1-4 drinks of beer per week	◯	◯	◯	◯	◯	◯
More than 4 drinks of beer per week	◯	◯	◯	◯	◯	◯
1-4 drinks of white wine per week	◯	◯	◯	◯	◯	◯
More than 4 drinks of white wine per week	◯	◯	◯	◯	◯	◯
1-4 drinks of red wine per week	◯	◯	◯	◯	◯	◯
More than 4 drinks of red wine per week	◯	◯	◯	◯	◯	◯
1-4 drinks of liquor per week	◯	◯	◯	◯	◯	◯
More than 4 drinks of liquor per week	◯	◯	◯	◯	◯	◯

36. Foods- Partner use

1. How do you think your partner eating the following types of food affects the success (getting pregnant and having a baby) of your IVF cycle?

	Very harmful	Harmful	No effect	Helpful	Very helpful	I don't know/ No opinion
No specific (regular) diet	◯	◯	◯	◯	◯	◯
Low fat diet	◯	◯	◯	◯	◯	◯
Low carbohydrate diet	◯	◯	◯	◯	◯	◯
Vegetarian diet	◯	◯	◯	◯	◯	◯
Organic food	◯	◯	◯	◯	◯	◯
Increase in fruit and vegetables	◯	◯	◯	◯	◯	◯
High protein diet	◯	◯	◯	◯	◯	◯
Full-fat milk or dairy products	◯	◯	◯	◯	◯	◯

37. Vitamins and herbal medicine- Partner use

1. How do you think that your partner taking the following vitamins affect the success (getting pregnant and having a baby) of your IVF cycle?

	Very harmful	Harmful	No effect	Helpful	Very helpful	I don't know/ No opinion
No vitamins or herbs	◯	◯	◯	◯	◯	◯
Prenatal vitamin	◯	◯	◯	◯	◯	◯
Mulitvitamin	◯	◯	◯	◯	◯	◯
Zinc	◯	◯	◯	◯	◯	◯
Dehydroepiandrosterone (DHEA)	◯	◯	◯	◯	◯	◯
Vitamin B6	◯	◯	◯	◯	◯	◯
Vitamin C	◯	◯	◯	◯	◯	◯
Vitamin D	◯	◯	◯	◯	◯	◯
Vitamin E	◯	◯	◯	◯	◯	◯
Chinese herbal medicine	◯	◯	◯	◯	◯	◯

38. Over the counter medications- Partner use

1. How do you think your partner's use of the following over the counter medications affect the success (getting pregnant and having a baby) of your IVF cycle?

	Very harmful	Harmful	No effect	Helpful	Very helpful	I don't know/ No opinion
No over the counter medications	◯	◯	◯	◯	◯	◯
Baby aspirin	◯	◯	◯	◯	◯	◯
Ibuprofen	◯	◯	◯	◯	◯	◯
Tylenol	◯	◯	◯	◯	◯	◯
Cold/allergy medication	◯	◯	◯	◯	◯	◯

39. Stress- Partner

1. How do you think that your partner's stress affects the success (getting pregnant and having a baby) of your IVF cycle?

	Very harmful	Harmful	No effect	Helpful	Very helpful	I don't know/ No opinion
No stress	◯	◯	◯	◯	◯	◯
Stress	◯	◯	◯	◯	◯	◯

40. Attitude- Partner

1. How do you think that your partner's attitude affects the success (getting pregnant and having a baby) of your IVF cycle?

	Very harmful	Harmful	No effect	Helpful	Very helpful	I don't know/ No opinion
Neither positive nor negative attitude	◯	◯	◯	◯	◯	◯
Positive attitude	◯	◯	◯	◯	◯	◯
Negative attitude	◯	◯	◯	◯	◯	◯

41. Body weight- Partner

1. How do you think the following partner's body weight affects the success (getting pregnant and having a baby) of an IVF cycle?

	Very harmful	Harmful	No effect	Helpful	Very helpful	I don't know/ No opinion
Underweight	◯	◯	◯	◯	◯	◯
Normal weight	◯	◯	◯	◯	◯	◯
Overweight	◯	◯	◯	◯	◯	◯
Obese	◯	◯	◯	◯	◯	◯

42. Yoga- Partner

1. How do you think different amounts of your partner doing yoga affects the success (getting pregnant and having a baby) of your IVF cycle?

	Very harmful	Harmful	No effect	Helpful	Very helpful	I don't know/ No opinion
No yoga	◯	◯	◯	◯	◯	◯
Once per week	◯	◯	◯	◯	◯	◯
2-6 sessions per week	◯	◯	◯	◯	◯	◯
7 or more sessions per week	◯	◯	◯	◯	◯	◯

43. Accupuncture- Partner

1. How do you think that your partner having accupuncture around the time of embryo transfer affects the success (getting pregnant and having a baby) of your IVF cycle?

	Very harmful	Harmful	No effect	Helpful	Very helpful	I don't know/ No opinion
No accupuncture	◯	◯	◯	◯	◯	◯
Accupuncture	◯	◯	◯	◯	◯	◯

44. Exercise- Partner

1. How do you think your partner's exercise affects the success (getting pregnant and having a baby) of your IVF cycle?

	Very harmful	Harmful	No effect	Helpful	Very helpful	I don't know/ No opinion
No exercise	◯	◯	◯	◯	◯	◯
Less than 1 hour per week	◯	◯	◯	◯	◯	◯
1-3 hours per week	◯	◯	◯	◯	◯	◯
4 or more hours per week	◯	◯	◯	◯	◯	◯

45. Rest- Partner

1. How do you think that your partner resting (avoiding exercise or strenuous activity) the week after the embryo transfer affects the success (getting pregnant and having a baby) of your IVF cycle?

	Very harmful	Harmful	No effect	Helpful	Very helpful	I don't know/ No opinion
Normal activities/ no rest	◯	◯	◯	◯	◯	◯
Limits on strenuous activities and exercise	◯	◯	◯	◯	◯	◯
Bed rest	◯	◯	◯	◯	◯	◯

46. Prayer- Partner

1. How do you think your partner praying for the success of your IVF cycle affects the success (getting pregnant and having a baby) of your IVF cycle?

	Very harmful	Harmful	No effect	Helpful	Very helpful	I don't know/ No opinion
No prayer	◯	◯	◯	◯	◯	◯
Prayer	◯	◯	◯	◯	◯	◯

47. Cell phones- Partner

1. How do you think that your partner's typical cell phone usage affects the success (getting pregnant and having a baby) of your IVF cycle?

	Very harmful	Harmful	No effect	Helpful	Very helpful	I don't know/ No opinion
Limiting cell phone usage	◯	◯	◯	◯	◯	◯
Typical cell phone usage	◯	◯	◯	◯	◯	◯

48. Environment/ plastics- Partner

1. How do you think that your partner's typical exposure to plastics (water bottles, food containers) affects the success (getting pregnant and having a baby) of your IVF cycle?

	Very harmful	Harmful	No effect	Helpful	Very helpful	I don't know/ No opinion
Limiting exposure to plastics	◯	◯	◯	◯	◯	◯
Typical exposure to plastics	◯	◯	◯	◯	◯	◯

49. Other factors- Partner

1. If there any other lifestyle factors (items or activities) that you think affect the success of your IVF cycle that have not been mentioned in this survey, please write them here.

50. Sources of information

1. How influential is each of the following in making your beliefs expressed in this survey?

	No effect	Influential	Very influential
Physician	◯	◯	◯
Nurse	◯	◯	◯
Friends	◯	◯	◯
Family	◯	◯	◯
Internet	◯	◯	◯
Books	◯	◯	◯

Other (please specify)

51.

1. If you have any other comments, please state them here.

52.

Thank you for your time!
